# Angioside: The role of Angiogenesis and Hypoxia in Lung Neuroendocrine Tumours According to Primary Tumour Location in Left or Right Parenchyma

**DOI:** 10.3390/jcm11195958

**Published:** 2022-10-09

**Authors:** Anna La Salvia, Raffaella Carletti, Monica Verrico, Tiziana Feola, Giulia Puliani, Massimiliano Bassi, Franz Sesti, Angelina Pernazza, Rossella Mazzilli, Giuseppe Lamberti, Alessandra Siciliani, Massimiliano Mancini, Chiara Manai, Federico Venuta, Mohsen Ibrahim, Silverio Tomao, Giulia D’Amati, Cira Di Gioia, Elisa Giannetta, Federico Cappuzzo, Antongiulio Faggiano

**Affiliations:** 1 Division of Medical Oncology 2, IRCCS Regina Elena National Cancer Institute, 00144 Rome, Italy; 2 Department of Translational and Precision Medicine, Sapienza University of Rome, 00185 Rome, Italy; 3 Department of Radiological, Oncological and Pathological Sciences, Sapienza University of Rome, 00185 Rome, Italy; 4 Department of Experimental Medicine, Sapienza University of Rome, 00185 Rome, Italy; 5 Neuroendocrinology, Neuromed Institute, IRCCS, 86077 Pozzilli, Italy; 6 Oncological Endocrinology Unit, IRCCS Regina Elena National Cancer Institute, 00144 Rome, Italy; 7 Department of Thoracic Surgery, Policlinico Umberto I, “Sapienza” University of Rome, 00161 Rome, Italy; 8 Department of Medico-Surgical Sciences and Biotechnologies, Sapienza University of Rome, 00185 Rome, Italy; 9 Department of Clinical and Molecular Medicine, Sant’Andrea Hospital, ENETS Center of Excellence, Sapienza University of Rome, 00185 Rome, Italy; 10 Department of Specialized, Experimental and Diagnostic Medicine, S. Orsola-Malpighi Hospital, University of Bologna, 40126 Bologna, Italy; 11 Department of Thoracic Surgery, Sant’Andrea Hospital, Sapienza University of Rome, 00185 Rome, Italy; 12 Division of Morphologic and Molecular Sant’Andrea Hospital, Sapienza University of Rome, 00185 Rome, Italy

**Keywords:** neuroendocrine tumours, lung NET, left side, angiogenesis, hypoxia, necrosis, prognostic factors

## Abstract

Well-differentiated lung neuroendocrine tumours (Lu-NETs), classified as typical (TC) and atypical (AC) carcinoids, represent 30% of NETs. Angiogenesis plays an essential role in NET development and progression. A higher vascular network is a marker of differentiation, with positive prognostic implications. Materials and Methods: We retrospectively evaluated microvessel density (MVD) by CD34 immunohistochemical (IHC) staining and hypoxia by IHC staining for Hypoxia-inducible factor 1α (HIF-1α), comparing right- and left-lung parenchyma in 53 lung NETs. Results: The median age was 66 years (39–81), 56.6% males, 24.5% AC, 40.5% left-sided tumours and 69.8% TNM stage I. The mitotic count was <2/10 per 10 HPF in 79.2%, and the absence of necrosis in 81.1%, 39.6% with Ki67, was ≤2%. The MVD, the number of vessels and the average vessel area median values were significantly higher in the right than the left parenchyma (*p*: 0.025, *p*: 0.019, *p*: 0.016, respectively). Hypoxia resulted present in 14/19 (73.6%) left tumours and in 10/20 (50%) right tumours in the parenchyma (*p*: 0.129). Conclusions: This study suggests a biological rationale for a different angiogenesis and hypoxia according to the Lu-NETs’ location. In our study, left primary tumours were less vascularized and most likely to present hypoxia than right primary tumours. This finding could have potentially useful prognostic and predictive implications for Lu-NETs.

## 1. Introduction

### 1.1. Lung Neuroendocrine Tumours: Classification, Features and Prognostic Factors

Neuroendocrine neoplasms (NENs) are a heterogeneous group of tumours originating from neuroendocrine cells, whose incidence has been significantly increasing every year [1]. NENs are highly heterogeneous tumours, and their classification criteria and prognoses vary according to their presence in different organs [2]. Lung NENs comprise approximately 20% of lung cancers [3]. According to the 2021 WHO classification of thoracic tumours [4], ‘neuroendocrine tumour (NET)’ is the proposed term to unify the nomenclature that includes well-differentiated grade 1 (G1) NETs and grade 2 (G2) NETs, which generally correspond, respectively, to typical (TC) and atypical (AC) carcinoids. Highly aggressive NENs encompass large-cell neuroendocrine carcinoma (LCNEC) and small-cell carcinoma (SCLC) [5,6]. TC and AC are rare neoplasms, accounting for about 1–2% of all lung neoplasms and about 25–30% of all well-differentiated NETs throughout the body [7]; SCLC and LCNEC account for approximately 20% and 3%, respectively, of lung carcinoma and about 90% of poorly differentiated neuroendocrine carcinoma of all origins [8,9,10]. TC and AC exhibit heterogeneous morphological, immunohistochemical and molecular characteristics and show different clinical and biological aggressiveness with different prognoses [11,12,13,14]. TC are more common, indolent and demonstrate lower relapse rates than AC, which are a more variegated group with unpredictable clinical behaviour [4]. Patients with central carcinoids may present with symptoms related to bronchial obstruction, including cough, wheezing, haemoptysis and recurrent pneumonia. Peripheral carcinoids are generally asymptomatic and are typically detected as incidental findings on chest imaging. Symptoms related to hormone secretion are rare. According to the current guidelines, histological diagnosis of carcinoids requires a proliferation of neuroendocrine cells that are uniform in shape with a fine granular chromatin, with indistinct nucleoli and abundant cytoplasm, and that are growing in an organoid, trabecular, insular, palisading or rosette-like arrangement [4,5]. Oncocytic, clear cells and melanin-laden carcinoids can occur. Based on the mitotic count and the presence of necrosis, carcinoid tumours are divided into typical and atypical: atypical carcinoids have 2 to 10 mitoses per 2 mm^2^ or necrosis, while typical carcinoids lack necrosis and have less than 2 mitoses per 2 mm^2^. Carcinoid tumours are positive for low-molecular-weight cytokeratins and are strongly reactive for neuroendocrine markers such as chromogranin A, synaptophysin, CD56 and INSM-1. TTF-1 tends to be positive in peripheral tumours but negative in central tumours. The proliferation marker Ki-67 is not part of the diagnostic criteria for lung carcinoids, particularly in the distinction between typical and atypical carcinoids [15]. Although tumours with a Ki-67 index > 5% are more likely to be AC, this is not an absolute cut-off value [4,16,17]. TNM staging is inadequate to stratify lung NET patients. Some works have suggested integrating pathological and molecular features in TNM staging [18,19]. Surgical treatment has long been considered the treatment of choice for lung carcinoids. Okereke et al., in a retrospective study, described 5- and 10-year overall survival (OS) rates of 96% and 88% for TC and 87% and 69% for AC after surgical resection [20]. Similar results were reported by Stolz, Lee, Zhong and Filosso [21,22,23,24].

In our study, we retrospectively analysed consecutive cases with histological diagnoses of primary lung carcinoids to suggest a biological rationale for a different angiogenesis according to the tumour side: the left vs. the right lung.

### 1.2. Angiogenesis, Hypoxia and NET

Angiogenesis is one of the hallmarks of cancer as it plays a key role in providing oxygen and nutrients for tumour cell growth and progression [25]. Targeting angiogenesis has been successfully explored as a therapeutic strategy in a wide spectrum of solid tumours. NENs are typically vascularized tumours, and it is well-known that the pathways regulating angiogenesis play an essential role in the development and progression of the disease [26]. It has been described that NENs over-express both the endothelial vascular growth factor (VEGF) and its tyrosine kinase receptors (VEGFR) in a high percentage of cases (64–80%) [26]. Other growth factors, such as the stem-cell factor receptor (c-kit), the platelet-derived growth factor (PDGF) and its receptors (PDGFR-α, PDGFR-β and PDGFR-αβ), have also been linked to the development and progression of NENs [27]. Hypoxia-inducible factor (HIF) is a transcriptional factor that responds to low oxygen levels. The dysregulation of HIF is vital for the formation of blood vessels in cancer, thereby accelerating cancer progression. Several lines of evidence show that the dense vascular network associated with low-grade NETs is more likely to be a marker of differentiation than a marker of aggressiveness, as opposed to what is observed in other epithelial tumours [26,28]. This phenomenon represents the so-called ‘neuroendocrine paradox’, meaning that vascularization is inversely related to the aggressiveness of the disease. However, other studies have observed that in VEGF and other angiogenic markers (such as ANG2 and PROK2), over-expression was correlated with a worse clinical outcome in patients with NENs [28,29,30,31,32]. Based on this strong rationale, many clinical trials have been developed to evaluate the activity of different anti-angiogenic agents, including monoclonal antibodies directed against VEGF (such as bevacizumab) and small molecules inhibiting tyrosine kinase activity (TKI) of receptors such as VEGFR and PDGFR [33]. Sunitinib, a multiple anti-angiogenic TKI, was approved in 2010 by health authorities (FDA and EMA) for the treatment of pancreatic NETs [34]. More recently, the phase III SANET-ep trial (NCT02588170; https://clinicaltrials.gov/ct2/show/NCT02588170 (accessed on 4 July 2022)) evidenced a significant improvement in patients’ outcome for the anti-angiogenic surufatinib versus placebo in extra-pancreatic NENs (including in the lung) [35]. Additionally, a phase II/III, randomized, double-blind study, the AXINET trial, demonstrated a significantly higher response rate for the anti-angiogenic axitinib for patients with advanced NENs of non-pancreatic origin, including the lung (NCT01744249) [36].

However, to date, a deep molecular profile of the angiogenetic key gene expression has not been elucidated, and, therefore, an optimal selection of patients for this approach is not possible. Interestingly, a retrospective analysis carried out by our group [37] identified a significant difference in necrosis in left-sided versus right-sided lung NENs, providing a rationale for a differential expression of angiogenesis and hypoxia according to the primary side of the tumour (right-sided versus left-sided), with potentially relevant implications.

With this study, we explored and compared angiogenesis and hypoxia, evaluated through immunohistochemistry, in NETs located in left- and right-lung parenchyma to establish if a difference exists with potential biological and prognostic implications.

## 2. Materials and Methods

### 2.1. Patient Population

We retrospectively collected all consecutive cases with lung carcinoid histological diagnoses from Sant’Andrea Hospital, ENETS Center of Excellence and the NETTARE Unit of the Policlinico Umberto I of Sapienza University of Rome from January 2014 to April 2021. The inclusion criteria were the histologically confirmed diagnoses of lung carcinoids, both TC and AC, according to the 2021 WHO classification [4]. All patients presented a surgically resected tumour, which was fixed in 10% neutral buffered formalin immediately after collection and underwent histological examination. Extra-pulmonary NETs and poorly differentiated lung NENs were excluded.

Data including age; gender; risk factors, in particular a tobacco smoking habit; left or right lung side; TNM stage (tumour extension, nodal involvement); and date of clinical diagnosis and of surgery were collected retrospectively. Progression-free survival (PFS) and overall survival (OS) were calculated in months.

### 2.2. Histopathological and Immunohistochemical Examination

For each included case, the pathologists of both Sapienza Hospitals revised microscopically the histological slides performed from all surgically resected tumours and stained them with haematoxylin–eosin, chromogranin A, synaptophysin, TTF-1 and Ki-67 at light microscopy to confirm the diagnoses according to the 2021 WHO criteria (organoid, trabecular or insular pattern of grown; positivity of neuroendocrine markers Chromogranin A, Synaptophysin and TTF-1; mitotic count; necrosis; Ki-67 proliferation index), and when the diagnoses were confirmed, they were divided into typical (<2 mitoses per 2 mm^2^, no necrosis, Ki-67 < 5%) and atypical carcinoid (2–10 mitoses per 2 mm^2^, necrosis, Ki-67 range 5–20%). The mitotic count was expressed as mitoses/10 HPF (high-power fields, 2 mm^2^); the Ki-67 index was obtained by counting the positive tumour cells in areas of higher nuclear labelling (so-called hotspots) and was expressed as a percentage.

For each case, immunohistochemical (IHC) stains on consecutive paraffin-embedded tissue sections (thickness 3 mm) were also performed at Policlinico Umberto I immunohistochemistry laboratory with the CD34 antibody, a specific marker for endothelial cells, and with the hypoxia-inducible factor 1α (HIF-1α) antibody, an oxygen-dependent subunit of the transcription factor, which plays a central role in tumour angiogenesis under hypoxic conditions.

The CD34 IHC stains were made using the BOND-III automated IHC stainer (Leica Biosystems, Milan, Italy), with the CD34 ready-to-use antibody (Novocastra, Newcastle upon Tyne, UK) and the HRP-DAB detection system; all immunostained slides were captured with the Aperio Scanner (Leica Biosystems, Milan, Italy), and twenty randomly selected images at 20× magnification for each slide were used to value the intra-tumoural number of vessels, the average vessel area and the microvessel density (MVD) by a computerized imaging software (Image J, NIH, Bethesda, MD, USA). The vessel area was expressed as µm² and the MVD as vessel number/total area.

The HIF-1α immunostaining was performed, after previous treatment in a microwave (750 W) with citrate buffer (0.01 mol/L, pH 6), by incubation with anti-HIF-1α rabbit polyclonal antibody (1:100 at room temperature; NB100–134; Novus Biologicals, Liddleton, CO, USA), using the UltraTek HRP Staining System (ScyTeK Laboratories, 205 South 600 West, Logan, UT 84321, USA) and 3,3′-diaminobenzidine (DAB) (Dako, Glostrup, Produktionsvej 42 Glostrup, 2600, Denmark). A negative control was obtained by omitting the primary antibody. For each immunostaining, two independent pathologists (CDG and RC) counted the positive neoplastic cells in 20 randomly selected, non-overlapping fields at 40×; only tumour cells with diffuse, brownish stained nuclei were considered positive. All carcinoids with ≥1% positive neoplastic cells were considered positive in HIF-1α immunostaining [38].

This study protocol has been approved by the review board of Sapienza University of Rome (reference number 5917).

### 2.3. Statistical Analysis

Categorical variables were expressed as number (percentage). Continuous variables were presented as median (range). PFS and OS were estimated using the Kaplan–Meier method; PFS was calculated from the date of surgery to the date of first progression according to the RECIST criteria v1.1, or disease-related death for PFS, and OS to the date of death, or last follow-up for OS. We performed the Chi-square test to identify associations between different variables. A *p*-value of <0.05 was considered statistically significant. The Kolmogorov–Smirnov test was applied to compare angiogenesis values between left and right parenchyma. Univariate analyses were performed using a Cox regression test for each variable of interest in order to detect the impact on PFS as well as on OS for each variable. For continuous parameters, the threshold was defined as the median value of the population. Multivariate analyses using a Cox proportional hazards regression analysis were performed to identify factors independently associated with prognosis. The results from the survival analyses were presented with the effect estimates, hazard ratios (HR) and 95% confidence interval [95% CI]. All statistical analyses were performed using IBM-SPSS version 25 (IBM Corporation, New York, NY, USA).

## 3. Results

### 3.1. Patient Characteristics

Fifty-three patients with histologically confirmed diagnoses of primary lung NETs were included in this study. The characteristics of the study population are summarized in Table 1. Overall, the median age of our patients was 66 years (39–81). We included 30 males (56.6%), 14 smokers (26.4%), 40 TC (75.5%) and 13 AC (24.5%); notably, 37 patients presented with a TNM stage I (69.8%). Nodal status was positive in five cases (9.4%). None of the included patients presented with functional hormonal syndromes associated with the tumour. Significant comorbidities, specifically disorders affecting the respiratory tract as well as those of the immune system, were absent for all the patients considered. Regarding the pathological features, the mitotic count was <2 per 2 mm^2^ in 42 cases (79.2%); necrosis was present in 10 cases (18.8%); and the Ki-67 index was ≤2% in 19 cases, between 3 and 19% in 27 cases (50.9%) and ≥20% in one case (1.8%). The Ki-67 index was not available for six patients (11.3%). Synaptophysin, chromogranin A and TTF-1 were positive in 92.5%, 75.5% and 47.2% of cases, respectively. The median follow-up was 23 months (0.7–323); 2-year and 5-year PFS rates were 100% and 93.8%, respectively; and 2-years and 5-years OS rates were both 96.2%.

According to primary tumour location, 23 (40.5%) were left-sided tumours, and the remaining tumours (*n* = 30) were located in the right-lung parenchyma.

Among the 23 left-sided lung NETs, 8 were males (34.8%), 9 cases (39.1%) presented an age ≤ the median value (66 years) and 9 patients were smokers (39.1%). Only 1 case presented a positive nodal status (4.3%), and 16 cases were diagnosed with a TNM stage I disease (69.6%). There were 8 cases with AC (34.8%), the mitotic count was <2 per 2 mm^2^ in 16 patients (69.6%), Ki-67 was ≤2% in 11 cases (47.8%) and necrosis was found in 7 cases (30.4%). Synaptophysin, chromogranin A and TTF-1 were positive in 17 (73.9%), 22 (95.6%) and 12 cases (52.1), respectively.

Among the 30 right-sided lung NETs, 15 were males (50.0%), 18 cases (60.0%) presented an age ≤ the median value and 10 patients were smokers (33.3%). Four cases presented a positive nodal status (13.3%), and twenty-one cases were diagnosed with a TNM stage I disease (70.0%). There were 5 cases with AC (16.7%), the mitotic count was <2 per 2 mm^2^ in 26 patients (86.7%), Ki-67 was ≤2% in 10 cases (33.3%) and necrosis was found in 3 cases (10.0%). Synaptophysin, chromogranin A and TTF-1 were positive in 23 (76.7%), 27 (90.0%) and 13 cases (43.3%), respectively.

### 3.2. Angiogenesis and Hypoxia in Left vs. Right Parenchyma in the Analysed Cases

Only samples with available tumoural tissue (41/53, 19 left-sided and 22 right-sided tumours) were used for IHC assessment of angiogenesis as well as evaluating the MVD, the number of vessels and the average vessel area. In all cases, MVD (expressed as the ratio between the number of vessels and the surface in mm^2^), number of vessels and average vessel area (expressed in μm^2^), the median values were 217.69, 652.00 and 196.4, respectively. The three variables considered (the median values of MVD, number of vessels and average vessel area) were significantly higher in carcinoids located in right-lung (251,99, 759.00 and 202.11, respectively) than left-lung (195.21, 529.00, 182.53, respectively) parenchyma (*p* = 0.025, *p* = 0.019, *p* = 0.016, respectively) (Table 2).

In the available tumoural tissue samples, hypoxia was assessed by evaluating HIF-1α positivity by IHC staining (39/53 carcinoids, 19 left-sided and 20 right-sided tumours, 2 cases were not evaluated). Hypoxia was present in 14/19 (73.7%) tumours located in the left lung and in 10/20 (50%) tumours in the right lung. The association was not statistically significant, *p* = 0.129.

The absence of expression of the TTF-1 was associated with the presence of hypoxia (in 14/16, 87.5%, of TTF-1-negative cases, *p* = 0.012). Among hypoxia-negative cases, 11/13 (84.6%) were TTF-1 positive, whereas among hypoxia-positive cases, 10/24 expressed TTF-1.

Tumoural necrosis was found in 7/23 (30.4%) carcinoids in the left lung versus 3/30 (10.0%) in the right lung (*p* = 0.059).

**Figure 1:**
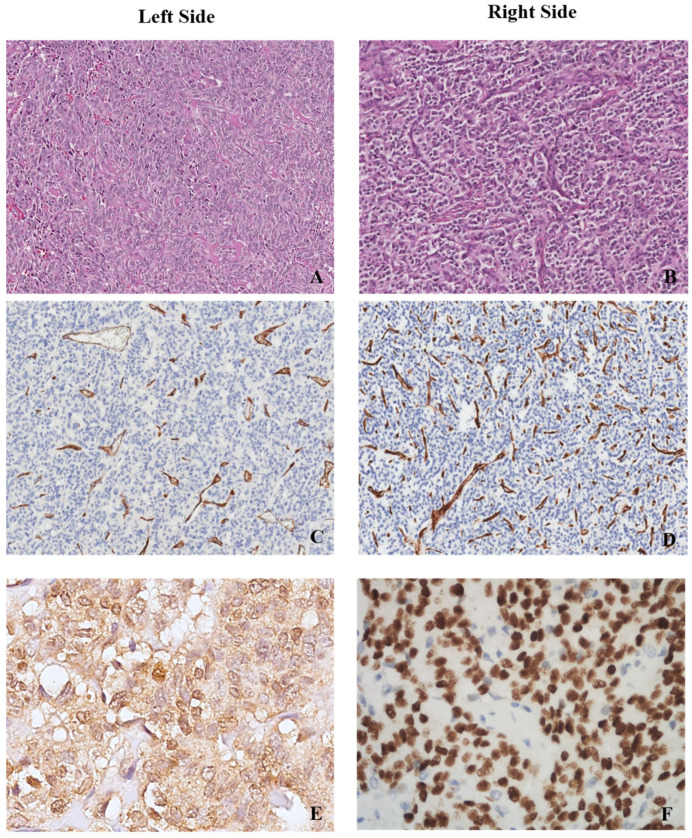
Primary lung carcinoid. (**A**) Right-sided lesion (**B**,**D**) showing a higher density in intratumoural vessels when compared to a left-sided case (**A**,**C**). (**A**,**B**) Haematoxylin and eosin stain, 10×, and (**C**,**D**) CD34 immunohistochemical stain for endothelial cells, 10×. A left-sided lung carcinoid with positivity for HIF-1α stain (**E**) and a right-sided lung case showing TTF-1 expression (**F**). (**E**) HIF-1α immunohistochemical stain, 40×, and (**F**) TTF-1 immunohistochemical stain, 40×.

The pathological findings, in terms of intratumoural vessel density, TTF-1 expression and HIF-1α positivity, of two illustrative cases (one right-sided and one left-sided) are depicted in Figure 1.

The multivariate models using a Cox proportional hazards regression analysis were not significant (*p* > 0.05) for all the variables of this study (age, gender, stage, diagnosis, presence of necrosis, mitotic count, Ki67, TTF-1 positivity, MVD, number of vessels, average vessel area and HIF-1α positivity).

### 3.3. Prognostic Impact of Side, Angiogenesis, Necrosis, TTF-1 and Hypoxia

Overall, the impact of angiogenesis, necrosis, TTF-1 and hypoxia resulted as not significant (*p* value higher than 0.05 for each variable considered).

Patients with right-lung carcinoids presented with a not statistically significant improvement in OS, unlike the ones with left-lung carcinoids, with a median OS of 308.00 months for right-lung NETs versus 102.44 months for left-lung NETs (*p* = 0.581, HR: 2.185, 95% CI 0.136–35.148).

The MVD, the number of vessels and the average vessel area, considering all analysed cases, did not show a significant impact on OS (*p* = 0.195, HR: 1.00, 95% CI 0.997–1.015; *p* = 0.195, HR: 1.00, 95% CI 0.999–1.005; *p* = 0.848, HR: 0.998, 95% CI 0.974–1.022, respectively).

Necrosis-positive cases presented a not statistically significant reduction in OS, unlike the tumours without necrosis, with a median OS of 102.400 months for necrosis-positive lung NETs versus 309.304 months for necrosis-negative lung NETs (*p* = 0.486, HR: 0.373, 95% CI 0.023–5.973).

The presence of hypoxia was associated with a shorter OS, with a 1-year OS rate of 90.9% versus 100% in hypoxia-negative cases. However, the impact on OS was not significant in the Cox regression analysis (*p* = 0.641, HR: 49.3, 95% CI 0.001–639,895,498.6).

TTF-1-negative cases presented a lower OS, with a 1-year OS rate of 90.0% versus 100% for TTF-1-positive cases. Notably, the difference resulted as more evident when comparing 10-years OS rates, with 60% for TTF-1 negative cases versus 100% for TTF-1 positive cases. However, the impact on OS was not significant in the Cox regression analysis (*p* = 0.468, HR: 0.14, 95% CI 0.001–1414.981).

## 4. Discussion

Angiogenesis is one of the hallmarks of cancer and has critical relevance to NETs, given their nature of highly vascularized tumours [39]. The angiogenetic process results from a complicated balance of pro- and anti-angiogenic factors produced by tumour cells and by cells of the tumour microenvironment (pericytes, mesenchymal, endothelial or immune cells). In the last few decades, angiogenesis has been identified as a therapeutic target for a wide range of solid tumours, including NETs. Several anti-angiogenic drugs have been investigated in the metastatic setting, such as sunitinib, sorafenib, bevacizumab, pazopanib, cabozantinib and lenvatinib [40,41,42,43,44]. To date, sunitinib is the only approved anti-angiogenic drug for advanced pancreatic NETs [34], even though a large amount of encouraging data regarding new agents, such as surufatinib and axitinib, are arising [45,46]. Both surufatinib and axitinib have proven to be active and safe for NET patients of extra-pancreatic origin (including lung primary origin) [35,36]. Many efforts have been made to identify predictive factors to personalize NET patients’ treatment, optimizing the use of anti-angiogenic agents. However, no specific biomarkers have been validated so far in this context.

MVD has been identified as a potential angiogenesis-related biomarker for NETs. Several works demonstrate that higher MVD values are associated with less aggressive tumours [47,48]. In analogy to these literature data, in our series, cases located in the right-lung parenchyma and presenting higher MVD values correlate with a better OS.

Therefore, the identification of a different angiogenetic texture, with a reduced MVD, number of vessels and average vessel area in left- versus right-lung parenchyma, may pave the way for the identification of innovative predictive features to personalize the therapeutic strategy. No available literature data support this finding, and further studies with larger sample sizes and prospective designs should be performed to establish the potential value of this aspect.

The second aim of our study was the evaluation of hypoxia, through the IHC determination of HIF-1α expression, in the tumoural samples of lung NET patients. Hypoxia and the hypoxia-inducible factor-1 (HIF-1) pathway regulate the expression of genes critical for cell growth, angiogenesis and tumour progression [49]. Previous studies have shown that HIF-1α expression is significantly higher in more aggressive pancreatic NETs than in benign tumours [47]. In this analysis, HIF-1α expression is significantly associated with poor tumour differentiation, higher proliferation index, presence of necrosis and lower MVD. These results were confirmed in our experience, with an increased positivity of HIF-1α in left- versus right-lung NETs, suggesting that a lower vascularization in left-lung tumours is associated with a higher presence of hypoxia and necrosis. Additionally, in the reported study [47], the presence of HIF-1α expression correlates with shorter survival, although this is not statistically significant (*p* = 0.06). This finding is similar to what we observed in our experience. Everolimus is a derivate of rapamycin which inhibits the mTOR serine/threonine kinase. Everolimus specifically inhibits mTORC1, disrupting the function of *p*-S6K1 and 4EBP1, the two main downstream effectors of mTORC1. It has been demonstrated that this effect interferes with mRNA translation of genes involved in cell cycle regulation and cellular response to hypoxia [50]. There have been several reports linking mTOR and HIF1α expression. The link between mTOR and HIF1α is based largely on the sensitivity of HIF1α to rapamycin. In this context, it has been postulated that HIF1α determines sensitivity to mTOR inhibitors [51]. This issue deserves further prospective analysis and validation in the field of lung NET.

The thyroid transcription factor (TTF-1) is a well-known biomarker for lung neoplasms, above all for adenocarcinomas [52] and for poorly differentiated lung neuroendocrine carcinomas [53]. For lung NETs, TTF-1 expression in carcinoids is considered specific when compared with those of extra-pulmonary origin [54]. However, the diagnostic value of this biomarker is low, given the high variability in its expression [55,56]. TTF-1’s role as a prognostic biomarker is still uncertain [57]. In our analysis, TTF-1 positivity was associated with patients’ outcomes. Notably, the absence of TTF-1 expression was significantly associated with the presence of hypoxia and a dismal prognosis. Interestingly, a previous translational study demonstrated that TTF-1 positively regulates VEGF and the major signalling receptor for VEGF as VEGFR2 lung cancer epithelial cells [58].

## 5. Limits

The current study presented some major limitations. One limit was the retrospective nature of patients’ inclusion, the collection of patients’ data and of their analysis. A second limit was represented by the patients’ sample size, with a potential negative impact on the multivariate models and in the survival analysis. Furthermore, another limit was the percentage of missing data, above all, for pathological variables, such as the IHC stain for TTF-1 or the Ki-67 value (missing in seven and six cases, respectively) and for a few clinical variables (such as smoking habit, which was lacking in 24 cases).

## 6. Conclusions

These findings, for the first time, suggest the existence of a biological difference in lung NETs located in left- versus right-lung parenchyma that is related to differences in terms of angiogenesis, hypoxia and necrosis in the two sides. These angiogenesis- and hypoxia-related biomarkers could reveal a prognostic significance and help clinicians with a therapeutic strategy for this population of patients.

Finally, this study represents an intriguing starting point for the search for innovative predictive markers to maximize the clinical benefit of anti-angiogenetic drugs for lung NETs.

## Figures and Tables

**Table 1:** Patient characteristics.

Feature	(*n* = 53)	%
**Gender**		
Male	30	56.6
Female	23	43.4
**Age**		
Median	66	
SD	11.15	
Min.	39	
Max	81	
**Smoke**		
No	15	28.3
Yes	14	26.4
Unknown	24	45.2
**Side**		
Left	23	43.4
Right	30	56.6
**Diagnosis**		
TC	40 (25 right-sided, 15 left-sided)	75.5
	13 (5 right-sided, 8 left-sided)	
AC		24.5
**Nodal status**		
Negative	46	86.8
Positive	5	9.4
Unknown	2	3.8
**TNM Stage**		
I	37	69.8
II	9	17
III	3	5.7
Unknown	4	7.5
**Synaptophysin**		
Neg.	1	1.9
Pos.	49	92.5
Unknown	3	5.7
**Chromogranin A**		
Neg.	5	9.4
Pos.	40	75.5
Unknown	8	15.1
**TTF-1**		
Neg.	21	39.6
Pos.	25	47.2
Unknown	7	13.2
**Mitotic count < 2 per 2 mm^2^**		
No	42	79.2
Yes	11	20.7
**Necrosis**		
No	43	81.1
Yes	10	18.8
**Ki-67**		
1–2%	19	35.8
3–19%	27	50.9
≥20%	1	1.8
Unknown	6	11.3

AC: atypical carcinoid, TC: typical carcinoid, TTF-1: thyroid transcription factor-1, SD: standard deviation, TNM: “tumor”, “node”, “metastasis” staging.

**Table 2:** Angiogenesis determined by IHC expression of CD34-positive cells in terms of number of vessels, MVD and average vessel area.

	Left Primary Carcinoids (*n* = 19)			Right Primary Carcinoids (*n* = 22)			Left vs. Right (Median Value)
	Median	SD	Range	Median	SD	Range	*p* Value
**Number of vessels**	529.00	544.26	113.00–2214.00	759.00	461.11	192.00–1790.00	**0.019**
**MVD**	195.21	179.03	37.51–735.06	251.99	153.09	63.74–594.29	**0.025**
**Average vessel area**	182.53	118.17	106.25–549.00	202.11	66.80	82.55–330.34	**0.016**

MVD: microvessel density, SD: standard deviation.

## Data Availability

Data are available upon reasonable request by qualified researchers to the authors.
